# Organization and Evolution of Subtelomeric Satellite Repeats in the Potato Genome

**DOI:** 10.1534/g3.111.000125

**Published:** 2011-07-01

**Authors:** Giovana A. Torres, Zhiyun Gong, Marina Iovene, Cory D. Hirsch, C. Robin Buell, Glenn J. Bryan, Petr Novák, Jiří Macas, Jiming Jiang

**Affiliations:** * Department of Horticulture, University of Wisconsin-Madison, Madison, Wisconsin 53706; † Departmento de Biologia, Universidade Federal de Lavras, Lavras, MG 37200, Brazil; ‡ Department of Plant Biology, Michigan State University, East Lansing, Michigan 48824; § Genetics Programme, Scottish Crop Research Institute, Invergowrie, Dundee, DD2 5DA, United Kingdom; ** Institute of Plant Molecular Biology, Biology Centre ASCR, CZ-37005, Ceske Budejovice, Czech Republic

## Abstract

Subtelomeric domains immediately adjacent to telomeres represent one of the most dynamic and rapidly evolving regions in eukaryotic genomes. A common feature associated with subtelomeric regions in different eukaryotes is the presence of long arrays of tandemly repeated satellite sequences. However, studies on molecular organization and evolution of subtelomeric repeats are rare. We isolated two subtelomeric repeats, CL14 and CL34, from potato (*Solanum tuberosum*). The CL14 and CL34 repeats are organized as independent long arrays, up to 1-3 Mb, of 182 bp and 339 bp monomers, respectively. The CL14 and CL34 repeat arrays are directly connected with the telomeric repeats at some chromosomal ends. The CL14 repeat was detected at the subtelomeric regions among highly diverged *Solanum* species, including tomato (*Solanum lycopersicum*). In contrast, CL34 was only found in potato and its closely related species. Interestingly, the CL34 repeat array was always proximal to the telomeres when both CL14 and CL34 were found at the same chromosomal end. In addition, the CL34 repeat family showed more sequence variability among monomers compared with the CL14 repeat family. We conclude that the CL34 repeat family emerged recently from the subtelomeric regions of potato chromosomes and is rapidly evolving. These results provide further evidence that subtelomeric domains are among the most dynamic regions in eukaryotic genomes.

The terminal regions of eukaryotic chromosomes are organized as a specialized structure to protect and maintain the stability of the chromosomes. Telomeres in most eukaryotes consist of short, tandemly repeated DNA sequences, such as (TTTGGG)n in humans and (TTTAGGG)n in plants (Zakian 1995). The length of such telomeric repeats can be extended during cell cycles by telomerase, a telomere-specific reverse transcriptase. In contrast to the extensive research on telomeres, there is only limited information available about the DNA sequences and the function associated with subtelomeric regions, which are immediately adjacent to telomeres. Subtelomeric regions contain repetitive sequences or duplicated DNA fragments, which make cloning and sequencing difficult. Thus, subtelomeric regions have not been fully sequenced even among the most extensively sequenced eukaryotic genomes using the traditional clone-by-clone–based sequencing approach (Mizuno *et al.* 2006; Riethman *et al.* 2005).

The presence of long arrays of tandemly repeated satellite sequences is a common feature associated with subtelomeric regions of many eukaryotic chromosomes. Subtelomeric repeats have been reported in numerous plant and animal species (Kojima *et al.* 2002; Sharma and Raina 2005; Spence *et al.* 1998). However, the function of such subtelomeric repeats remains largely unknown. Plants in the Alliaceae family lack the typical (TTTAGGG)n telomeric DNA sequences that are almost universal in all other plants (Fuchs *et al.* 1995). Instead, the chromosomal ends of *Alliaceae* species consist of highly repetitive satellite repeats, which structurally resemble the subtelomeric repeats found in other plant species. These satellite repeats were proposed to replace the function of telomeric DNA in stabilizing the chromosomal ends (Pich *et al.* 1996). Recently, Jain *et al.* (2010) demonstrated that *Schizosaccharomyces pombe* could survive in the absence of telomerase. Interestingly, the chromosomes in surviving cells had amplified subtelomeric DNA derived from rDNA (Jain *et al.* 2010). These results suggest that subtelomeric repeats may provide a supplemental role in chromosome stability.

Although subtelomeric repeat sequences have been reported in numerous plant species. However, an in-depth study of the structure and organization of subtelomeric repeats has been done in only a few species (Cheng *et al.* 2001; Dechyeva and Schmidt 2006; Zhong *et al.* 1998). Most importantly, it remains largely unknown how these repeats emerged and evolved during evolution. We identified two subtelomeric repeats, CL14 and CL34, in potato (*Solanum tuberosum*). The structure and organization of these two repeats were studied using a combination of fluorescence *in situ* hybridization (FISH) and fiber-FISH techniques. We also report the evolutionary history of these two repeats in a set of diverged *Solanum* species.

## Materials and Methods

### Plant materials

A homozygous doubled monoploid (2n = 2x = 24) clone, DM1-3, which was developed from a diploid potato species *Solanum phureja* (Snider and Veilleux 1994), was used for 454 genome sequencing and cytogenetic studies. Five *Solanum* species, including *S. verrucosum* (A genome, PI 275260), *S. cardiophyllum* (B genome, PI 347759), *S. chromatophilum* (P genome, PI 365339), tomato (*S. lycopersicum*, cv MicroTom) (T genome), and *S. etuberosum* (E genome, PI 558288), were used for comparative FISH mapping of the telomeric and subtelomeric repeats. All these species are diploids with a chromosome number of 24. Seeds of all *Solanum* species, except for the tomato variety MicroTom, were obtained from USDA/ARS, Potato Introduction Station, Sturgeon Bay, WI.

### Bioinformatic identification of tandemly repeated potato sequences

A set of 1,238,463 whole genome shotgun 454 sequences derived from DM1-3 was subjected to similarity-based clustering analysis (Macas *et al.* 2007) to identify major families of repetitive sequences present in the potato genome. The average length of the 454 reads was 361 bp and the whole set represented 446.7 Mb of sequence data, corresponding to about 0.53× coverage of the potato genome (1C = 840 Mb) (Arumuganathan and Earle 1991). The clustering analysis was performed using a graph-based approach as described previously (Novak *et al.* 2010), employing read similarity cutoff of 90% over at least 55% of the longer sequence length. Reads within individual clusters were assembled and further investigated using a set of custom-made BioPerl and R scripts to find out which type and family of repeats they represent (Macas *et al.* 2007; Novak *et al.* 2010). Clusters containing satellite repeats were identified based on the presence of tandem subrepeats within their read or assembled contig sequences. These satellite repeats were characterized using oligomer frequency analysis of their 454 reads as described previously (Macas *et al.* 2010). The analysis was performed for oligonucleotides of length k = 17 or 20 and reconstruction of major sequence variants was done using a minimal seed k-mer frequency of 0.0001 and extension threshold of 10%.

### FISH and fiber-FISH

Mitotic and meiotic chromosomes were prepared following published protocols (Dong *et al.* 2000; Lou *et al.* 2010). An *A. thaliana* plasmid clone, pAtT4 (Richards and Ausubel 1988), was used as a telomeric DNA probe. Probes for the CL14 and CL34 repeats were prepared by PCR amplification from DM1-3 genomic DNA using primers designed according to the monomers sequences: 14F (5′CTGGGGCACATTTGACCTTC3′), 14R (5′ ATTTTGCCGATTTTCGTGTG3′), 34F (5′ ACGCCTTTTTGCTCATTCTTA3′), and 34R (5′CCCGGCTACCTTCAATCTTTA3′). The PCR was performed in 25 μl of reaction (1× PCR buffer, 3 mM MgCl_2_, 0.2 mM dNTPs, 0.2 μM primers, 0.5 U of Taq polymerase (Invitrogen, Carlsbad, California), 10 ng of template DNA) for 32 cycles of 30 sec at 95°C, 30 sec at 58°C and 45 sec at 72°C, preceded by initial denaturation (1 min at 95°C) and followed by a final extension step (5 min at 72°C). Probes were labeled with either biotin-16-UTP or digoxigenin-11-dUTP (Roche Diagnostics, Indianapolis, IN) using a standard nick translation reaction. FISH and fiber-FISH were conducted according to published procedures (Jackson *et al.* 1998; Jiang *et al.* 1995). Chromosomes were counterstained with 4’,6-diamidino-2-phenylindole (DAPI) in Vectashield antifade solution (Vector Laboratories, Burlingame, CA). The fluorescence signals were captured using a Hamamatsu CCD camera. The images were processed with Meta Imaging Series 7.5 software using an Olympus BX51 epifluorescence microscope. The final contrast of the images was processed using Adobe Photoshop CS3 software.

### Cloning of repeat junction and sequencing analysis of junction clones

The primers described above for the CL14 and CL34 repeats along with a telomeric DNA primer, Tel (5′GTTTAGGGTTTAGGGTTTAG3′), were used to amplify potential junctions between the three different repeats. The PCR reactions were carried out the same as for the FISH probe amplification, using a single primer or combinations of two primers (14F/34R, 34F/14R, 34R/Tel, 14R/Tel). Distinct bands less than 1000 bp were excised from agarose gels and the DNA was purified using a QIAquick gel extraction kit (Qiagen, Venlo, Netherlands). Purified DNA fragments were cloned using a TOPO TA cloning kit (Invitrogen). Positively transformed clones were selected using colony PCR and DNA was isolated using a QIAprep spin miniprep kit (Qiagen). Sequencing of positive clones was conducted using ABI Big Dye sequencing.

Obtained sequences were evaluated for quality and vector sequences were removed using Pregap4 version 1.5 in the Staden package (http://staden.sourceforge.net/). The junctions and structure of the repeats in the quality sequences was analyzed by aligning them with the consensus sequences of CL14, CL34, and telomeric repeats using Gap4 version 4.10 (Staden package) with manual refinements.

## Results

### Identification of two subtelomeric repeats in potato

**Figure 1:**
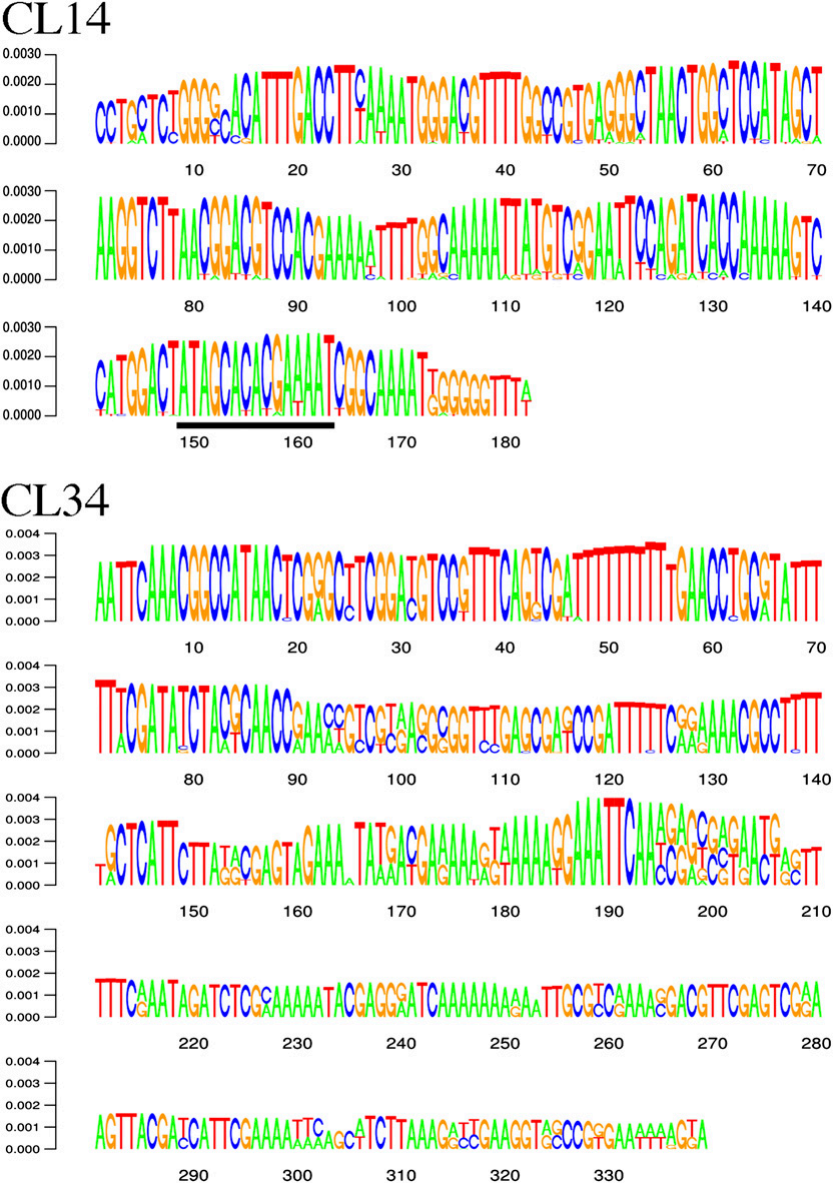
Consensus sequences of CL14 and CL34 repeats inferred from fragments reconstructed from the most frequent 17-mers (CL14) or 20-mers (CL34) detected in 454 sequence reads. The consensus is displayed as sequence logo where the height of the letters corresponds to frequencies of corresponding k-mers. Major sequence variants are displayed along with the prevailing bases. The CL14 region conserved in other *Solanum* and *Nicotiana* satellite repeats is underlined. Detailed information about reconstructed fragments used to create the consensus logos are provided as Figure S1.

Subtelomeric satellite repeats were identified by bioinformatic analysis of 1.24 million 454 Roche sequence reads generated from a homozygous diploid potato clone, DM1-3 (2n = 2x = 24), which was derived from chromosome doubling of a monoploid. DM1-3 has been sequenced by the Potato Genome Sequencing Consortium (http://www.potatogenome.net). The analysis was performed using a similarity-based 454 read clustering approach which allows *de novo* identification of all major types of genomic repeats (Macas *et al.* 2007; Novak *et al.* 2010). Resulting clusters of mutually overlapping reads corresponding to different repeat families were screened for the presence of tandem repeats and their chromosomal localization was investigated by FISH. Two repeats, CL14 and CL34, were found to be located at chromosome termini (see below). Based on 454 read counts in the clusters (5313 reads in CL14 and 2614 reads in CL34) the repeats were estimated to make up 0.4% and 0.2% of the DM1-3 genome, respectively. Due to large amounts of sequencing data available for these repeats their composition was investigated using an alignment-free approach employing oligomer frequency statistics (Macas *et al.* 2010). This analysis led to the identification of the most conserved sequence motifs and to reconstruction of consensus sequences for both repeats (Figure 1 and supporting information, Figure S1).

The CL14 repeat is composed of 182-bp monomers that are well homogenized in the potato genome based on the absence of distinct sequence variants among the most frequent reconstructed fragments (Figure S1). CL14 sequences do not share any detectable similarity to the CL34 repeat. Searching GenBank and PlantSat (Macas *et al.* 2002) databases revealed a high similarity to a subtelomeric tandem repeat from *Solanum brevidens* (88% identity over 147 bp) (Preiszner *et al.* 1994) and lower similarities scattered along its monomer sequence with related satellite repeats from *S. circaeifolium* and tomato (*S. lycopersicum*) (Schweizer *et al.* 1988; Stadler *et al.* 1995). In addition, a small part of the CL14 sequence was found to be present in a set of diverse *Nicotiana* satellite repeats including HRS, GRS and NPAL repeats (Gazdova *et al.* 1995; Koukalova *et al.* 2010; Matyasek *et al.* 1989). The similarity region varied in length among the different satellites, but it always included the region between positions 149-163 of the CL14 monomer (underlined in Figure 1). The CL34 repeat was characterized by predominant monomer size of 339 bp. In contrast to CL14, the CL34 sequences were more variable, including the presence of short direct subrepeats and various sequence subfamilies (Figure S1). Sequences similar with CL34 were only detected in two satellite repeats from *S. acaule* and *S. demissum* (Schweizer *et al.* 1988; Zanke and Hemleben 1997), both of these species being closely related to cultivated potato (Rodriguez *et al.* 2010).

### CL14 and CL34 repeats locate exclusively at the distal ends of potato chromosomes

**Figure 2:**
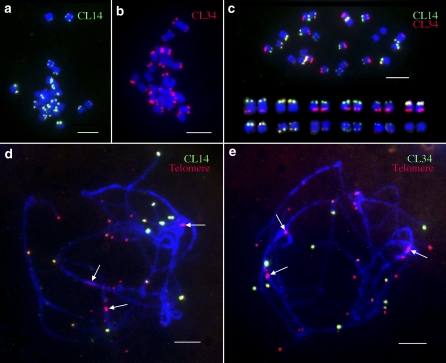
FISH mapping of CL14 and CL34 repeats in diploid potato clone DM1-3. (a) FISH mapping of CL14 on somatic metaphase chromosomes. (b) FISH mapping of CL34 on somatic metaphase chromosomes. (c) FISH mapping of both CL14 (green) and CL34 (red) on somatic metaphase chromosomes. Homologous chromosome pairs were identified based on FISH signal patterns and arranged according to their sizes. (d) FISH mapping of CL14 (green) and the telomeric DNA probe pAtT4 (red) on meiotic pachytene chromosomes. (e) FISH mapping of CL34 (green) and the telomeric DNA probe pAtT4 (red) on meiotic pachytene chromosomes. Arrows in (d) and (e) point to several interstitial signals derived from the telomeric DNA probe. Bars = 5 μm.

FISH analysis revealed that both CL14 and CL34 repeats locate exclusively at the distal ends of most DM1-3 chromosomes (Figure 2, a and b). The copy numbers of the two repeats at different chromosomal ends varied significantly based on the sizes and intensities of the FISH signals.

The subtelomeric locations of these two repeats were confirmed by co-hybridization with a telomeric DNA probe, pAtT4, on meiotic pachytene chromosomes (Figure 2, d and e). All of the CL14 and CL34 signals partially or completely overlapped with the telomeric DNA signals. Therefore, these two repeats are exclusively located at the end of the chromosomes. The pAtT4 probe, which was isolated from *Arabidopsis thaliana* (Richards and Ausubel 1988), generated several major FISH signals in interstitial chromosomal regions (Figure 2, d and e), confirming the previous reports that potato centromeric regions contain telomere-similar DNA sequences (Tek and Jiang 2004; Zhu *et al.* 2008).

**Figure 3:**
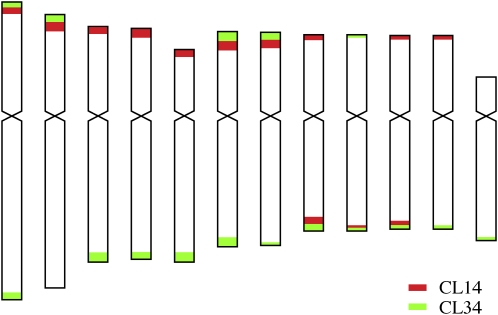
An ideogram illustrating the distribution of CL14 and CL34 repeats on potato chromosomes. This ideogram was generated based on the FISH information from Figure 2c.

When the CL14 and CL34 repeats were comapped on somatic metaphase chromosomes, several chromosomal ends hybridized to both repeats. It was not possible to determine the relative distal/proximal positions of the two repeats at the same chromosomal ends (Figure 2c). However, several CL34 loci were found to be distal to the CL14 loci at the same ends on pachytene chromosomes (Figure S2). Interestingly, CL34 tended to be dominant at the ends of the long arms, whereas CL14 more frequently dominated at the ends of the short arms (Figures 2c and 3). Only two of the 24 chromosomal ends lacked unambiguous CL14 or CL34 signals on somatic metaphase chromosomes (Figure 3). However, we cannot exclude the possibility of the presence of short arrays of CL14/CL34 repeats at these ends, which may be beyond the detection limit the FISH technique.

### CL14 and CL34 are organized as independent tandem arrays

Sequence analysis revealed no similarity between the CL14 and CL34 repeats, indicating that these elements are two unrelated repeat families. We conducted dual-color fiber-FISH using DNA fibers prepared from DM1-3. We did not observe any unambiguous intermingled fiber-FISH signals derived from both repeats. Thus, the CL14 and CL34 repeats are organized as independent arrays in the potato genome.

**Figure 4:**
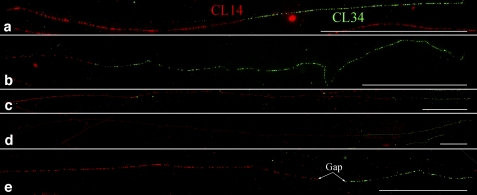
Fiber-FISH analysis of CL14 and CL34 repeats. (a–d) Representative fiber-FISH signals with different combinations of long and short CL14 (red) and CL34 (green) signals. The red and green signals appear to be directly connected on all four signals. (e) A fiber-FISH signal containing a clear gap between the CL14 and CL34 signals. Bars = 50 μm.

Direct connections between the CL14 and CL34 repeat arrays were frequently observed on fiber-FISH signals. We observed numerous combinations of long/short CL14 signals connected with long/short CL34 signals (Figure 4, a–d). These different combinations of long/short CL14/CL34 signals likely represent DNA loci derived from different chromosomal ends. A clear gap was observed at the CL14-CL34 junctions on some fibers (Figure 4e), suggesting the presence of other sequences at the junction between the two repeat arrays. However, a gap either too small (submicroscopic size) or too big (which would result in two “independent” signals) may not be detected by the fiber-FISH technique.

The longest observed CL14 and CL34 fibers were 1031 and 322 μm, respectively, representing approximately 3 Mb and 966 kb of DNA. Thus, a single CL14 or CL34 locus can account for up to 0.3% or 0.1% of the potato genome. The fiber-FISH results suggest that the percentages of these two repeats in the potato genome are much higher than the estimations based on the 454 sequence read counts. This discrepancy may be caused by the low genome coverage (0.5×) of the 454 sequencing data, which may lead to sequencing bias against repetitive DNA.

**Figure 5:**
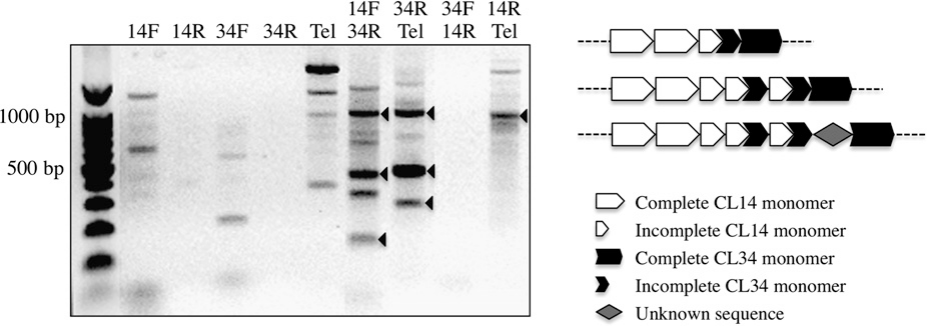
Cloning and characterization of the junctions among the telomeric and subtelomeric repeats. Left panel: PCR using primers designed from telomeric DNA (Tel), CL14 (14F and 14R), and CL34 (34F and 34R) repeats. PCRs using individual primers were performed to identify potential amplified fragments derived from the same primers. Arrowheads point to the PCR fragments that were cloned and sequenced. Right panel: Three patterns of CL14-CL34 junction sequences were found in the sequenced junction clones. Truncated CL14 and CL34 repeats and an unknown sequence of 30 bp were found at the junction regions. (Note that the schematic has not been drawn to scale.)

DNA primers specific to CL14 and CL34 were designed to amplify potential junctions between these two repeats. Polymerase chain reaction (PCR) using different combinations of these primers produced several distinct bands (Figure 5). Three PCR fragments corresponding to approximately 200 bp, 500 bp, and 1000 bp were cloned and sequenced. We sequenced 2, 19, and 5 clones, respectively, derived from these three PCR fragments. Sequence analysis revealed that the junctions between the CL34 and CL14 arrays were either a direct connection in different positions within the repeat monomer or a mixture of fragments of both repeats. Only one 30-bp unknown DNA sequence was found in the sequenced junction clones (Figure 5). These results confirmed the direct connection of the CL14 and CL34 arrays at some chromosomal ends.

### Association of CL14 and CL34 repeats With telomeric DNA

**Figure 6:**
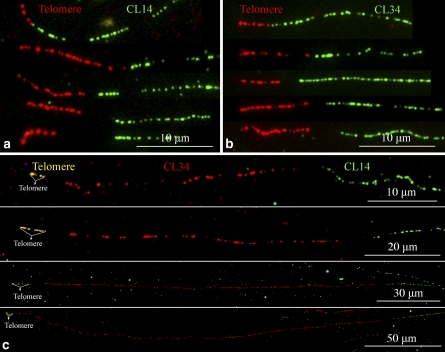
Fiber-FISH analyzing the association of CL14 and CL34 repeats with telomeric DNA. (a) Five representative fiber-FISH signals derived from probes pAtT4 (red) and CL14 (green). (b) Five representative fiber-FISH signals derived from probes pAtT4 (red) and CL34 (green). Only the most proximal part of each CL14/CL34 signal was included in each image. Note the different sizes of the telomeric DNA and gap in the junctions, indicating these signals may be derived from different chromosomal ends. (c) Fiber-FISH analysis of telomere (yellow), CL34 (red), and CL14 (green). The CL34 signals within the four images show significantly different sizes. Only the most proximal part of each CL14 signal was included in the image.

In order to reveal the association of the two subtelomeric repeats with telomeric DNA, we conducted fiber-FISH experiments using pAtT4 and CL14/CL34 as probes. Direct connections between pAtT4 and CL14/CL34 signals were observed on some DNA fibers (Figure 6, a and b). However, some fiber-FISH signals showed unambiguous gaps in the junction between telomeric DNA and the CL14/CL34 signals (Figure 6, a and b), suggesting the presence of additional DNA sequences at the junctions.

We then conducted fiber-FISH using three probes: CL34 (red), CL14 (green), and pAtT4 (yellow, by mixing 50% pAtT4 DNA in red and 50% pAtT4 DNA in green). In all fiber-FISH signals that contain all three repeats, the CL34 repeat array always localized between telomeric DNA and a CL14 repeat array (Figure 6c). These results agree with the observed distal positions of CL34 repeats relative to CL14 repeats on the pachytene chromosomes (Figure S2). We analyzed a total of 38 telomere-CL34-CL14 fiber-FISH signals. The CL34 arrays within these signals can be grouped into five size categories with average sizes of 34.9 ± 0.6 μm (n = 4), 80.3 ± 0.9 μm (n = 20), 146.2 ± 6.0 μm (n = 5), 198.1 ± 1.0 μm (n = 5) and 289.6 ± 12.0 μm (n = 4), respectively, which may represent DNA loci at different chromosomal ends.

PCR using a telomeric DNA primer (Tel) with either CL14 or CL34-specific primers produced several distinct bands. We cloned three PCR fragments (308 bp, 481 bp, and 1013 bp) derived from the Tel/34R primer pair and one PCR fragment (940 bp) from the Tel/14R primer pair (Figure 5). Sequencing and analysis of a total of 22 plasmid clones revealed that the CL14 and CL34 repeats were all directly connected with the telomeric repeats within these junction clones, although different incomplete CL14/CL34 monomers were found at the junctions.

### Divergence of CL14 and CL34 repeats among *Solanum* species

FISH mapping of CL14 and CL34 repeats was performed on metaphase chromosomes of several *Solanum* species, including tomato (*S. lycopersicum*), which has diverged from potato for approximately 7 million years (Nesbitt and Tanksley 2002), and *S. etuberosum*, which is more distantly related to potato than tomato is to potato. *S. verrucosum* (A genome species), a possible progenitor of cultivated potato (Rodriguez *et al.* 2010), is the closest relative to potato among the species we investigated. *S. cardiophyllum* (B genome species) and *S. chromatophilum* (P genome species) are more distantly related cultivated potato than *S. verrucosum*.

**Figure 7:**
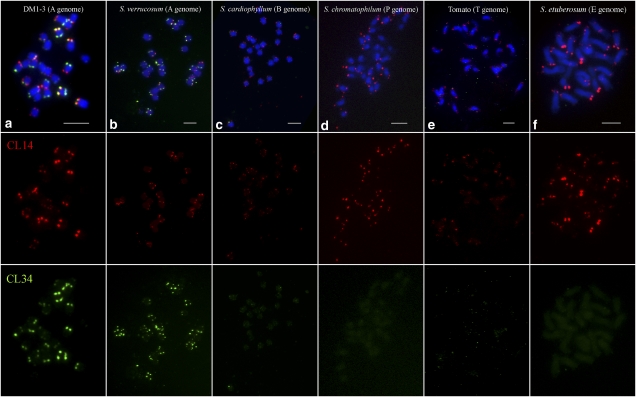
FISH mapping of CL14 and CL34 repeats among six different *Solanum* species. (a) DM1-3 potato (A genome). (b) *S. verrucosum* (A genome). (c) *S. cardiophyllum* (B genome). (d) *S. chromatophilum* (P genome). (e) Tomato (T genome). (f) *S. etuberosum* (E genome). The red (CL14) and green (CL34) color channels were digitally separated for better visualization of the FISH signals. Bars = 5 μm.

Both CL14 and CL34 showed similar hybridization patterns on chromosomes of *S. verrucosum* compared with DM1-3 chromosomes (Figure 7, a and b). Overall, the CL14 repeat showed a similar hybridization pattern among all *Solanum* species, although few nonsubtelomeric FISH signals were observed on chromosomes in several species (Figure 7, b and c). Thus, the CL14 repeat has largely maintained its subtelomeric locations among *Solanum* species that have diverged for more than 7 million years. However, the CL34 repeat generated very faint subtelomeric signals in *S. cardiophyllum* (Figure 7c) and tomato (Figure 7e) and no unambiguous signals (only very faint background signals) in *S. chromatophilum* (Figure 7d) and *S. etuberosum* (Figure 7f). These results show that the CL34 repeat has emerged recently and been amplified in potato and its close relatives.

## Discussion

### Organization of subtelomeric satellite repeats in plants

Cytologically confirmed subtelomeric satellite repeats have been reported in a number of plant species, including rice (Cheng *et al.* 2001), tomato (Lapitan *et al.* 1989), maize (Li *et al.* 2009), barley (Brandes *et al.* 1995), tobacco (Chen *et al.* 1997; Kenton *et al.* 1993), rye (Vershinin *et al.* 1995), *Silene latifolia* (Buzek *et al.* 1997), and *Beta* species (Dechyeva and Schmidt 2006). The subtelomeric locations of these repeats were confirmed mostly by *in situ* hybridization on somatic metaphase chromosomes. In some studies, the close physical association of these repeats with telomeres was demonstrated by either fiber-FISH (Cheng *et al.* 2001; Zhong *et al.* 1998; Dechyeva and Schmidt 2006; Sykorova *et al.* 2003) or by repeat junction cloning and sequencing (Fajkus *et al.* 1995; Sykorova *et al.* 2003). We demonstrated that both CL14 and CL34 repeats locate very close to the telomeric ends of potato chromosomes. At least some of the CL14 and CL34 loci are directly connected with telomeric (TTTAGGG)n repeats (Figure 5). Currently, we cannot exclude the possibility that additional subtelomeric repeats exist in the potato genome. Thus, some of the large gaps between telomere and CL14/CL34 sequences may be occupied with such additional repeats, which may be not as abundant as the CL14/CL34 families and thus were not found in the informatics based survey.

Multiple subtelomeric repeats have been reported in some plant species (Brandes *et al.* 1995; Chen *et al.* 1997; Dechyeva and Schmidt 2006; Vershinin *et al.* 1995). Traditional gel blot hybridizations can hardly be used to analyze the organization and relative positions of multiple repeats in a genome. Using a multicolor fiber-FISH approach, we demonstrated that the unrelated CL14 and CL34 repeats are organized into independent but tightly linked arrays. If one of the repeats was derived from the other repeat, and the two repeats had coevolved, then it would be expected to detect intermingled fiber-FISH signals derived from both repeats (Adawy *et al.* 2004). Fiber-FISH also revealed that the CL34 repeats appear to be always proximal to the telomere when a chromosomal end contains both CL14 and CL34 arrays (Figure 6c).

### Dynamics of the DNA sequences in subtelomeric regions

Presence of long arrays of satellite repeats makes it difficult to track the evolution of DNA sequences located in subtelomeric regions. However, the subtelomeric regions contain only single or low copy DNA sequences in some species. The subtelomeric regions of human chromosomes contain a mosaic patchwork of segmentally duplicated DNA blocks that are often present near the ends of multiple chromosomes (Riethman *et al.* 2005). Some of these segmental duplications contain transcribed genes, thus making the duplications a potential source of phenotypic diversity (Linardopoulou *et al.* 2005). Extensive studies of subtelomeric sequences in the human genome suggest that these regions are extraordinarily dynamic and are possibly involved in frequent sequence exchanges between ends of nonhomologous chromosomes (Linardopoulou *et al.* 2005).

The subtelomeric regions of *A. thaliana* chromosomes also lack major satellite repeats. The variation of a 3.5-kb DNA sequence adjacent to the telomere of the short arm of chromosome 1 was studied in 35 *A. thaliana* accessions (Kuo *et al.* 2006). This region showed dynamic sequence changes and rearrangements with characteristics of nonhomologous end-joining (NHEJ) events associated with double-strand break (DSB) repair. Such NHEJ events were also detected in the subtelomeric regions of the right arm of chromosome 3 (Wang *et al.* 2010). Thus, NHEJ events may play an important role in generating the sequence variation associated with the subtelomeric regions of *A. thaliana* chromosomes.

Our comparative FISH analysis of the CL14 and CL34 repeats in a diverse set of *Solanum* species provide additional strong evidence for the dynamic nature in the subtelomeric regions immediately adjacent to the telomeres. CL14 is an ancient repeat that has maintained its subtelomeric positions in all *Solanum* species (Figure 7) and its sequence appears to be related to a number of mostly subtelomeric satellite repeat families in *Solanum* and *Nicotiana* species. In contrast, CL34, a relatively new repeat, has been amplified in cultivated potato as well as in its most closely relatives. Interestingly, fiber-FISH analysis showed that the CL34 loci are more proximal to the telomeres than CL14 loci on the same chromosome ends (Figure 6c). Thus, the dynamic rearrangements and sequence exchanges between nonhomologous chromosome ends can result in the formation and amplification of new satellite repeat families or in sequence diversification of existing ones as was shown for subtelomeric arrays of VicTR-B satellites in *Vicia grandiflora* (Macas *et al.* 2006). High frequency of sequence rearrangements acting close to the telomeres could also explain the observed higher heterogeneity of CL34 monomers compared to CL14 monomers.

## Supplementary Material

Supporting Information
