# Supplementary material for: Organization and Evolution of Subtelomeric Satellite Repeats in the Potato Genome
Source: G3 (Bethesda). 2011 Jul 1;1(2):85–92. doi: 10.1534/g3.111.000125 (PMC3276127; doi:10.1534/g3.111.000125)
Supplement: Supporting Information [file supp_1_2_85__index.html]

Supporting Information 

# Organization and Evolution of Subtelomeric Satellite Repeats in the Potato Genome

## Supporting Information for Torres *et al.*, 2011

**Files in this Data Supplement:**

- Supporting Information - Figures S1 and S2 (PDF, 804 KB)
- Figure S1 - Mutual positions and sequences of fragments reconstructed from the most frequent k-mers used to build consensus sequences of the CL14 and CL34 repeats (PDF, 388 KB)
- Figure S2 - FISH mapping of CL14 (green) and CL34 (red) on pachytene chromosomes of DM1-3 (PDF, 420 KB)
