# Supplementary material for: Organization and Evolution of Subtelomeric Satellite Repeats in the Potato Genome
Source: G3 (Bethesda). 2011 Jul 1;1(2):85–92. doi: 10.1534/g3.111.000125 (PMC3276127; doi:10.1534/g3.111.000125)
Supplement: Supporting Information [file supp_1.2.85_000125SI.pdf]

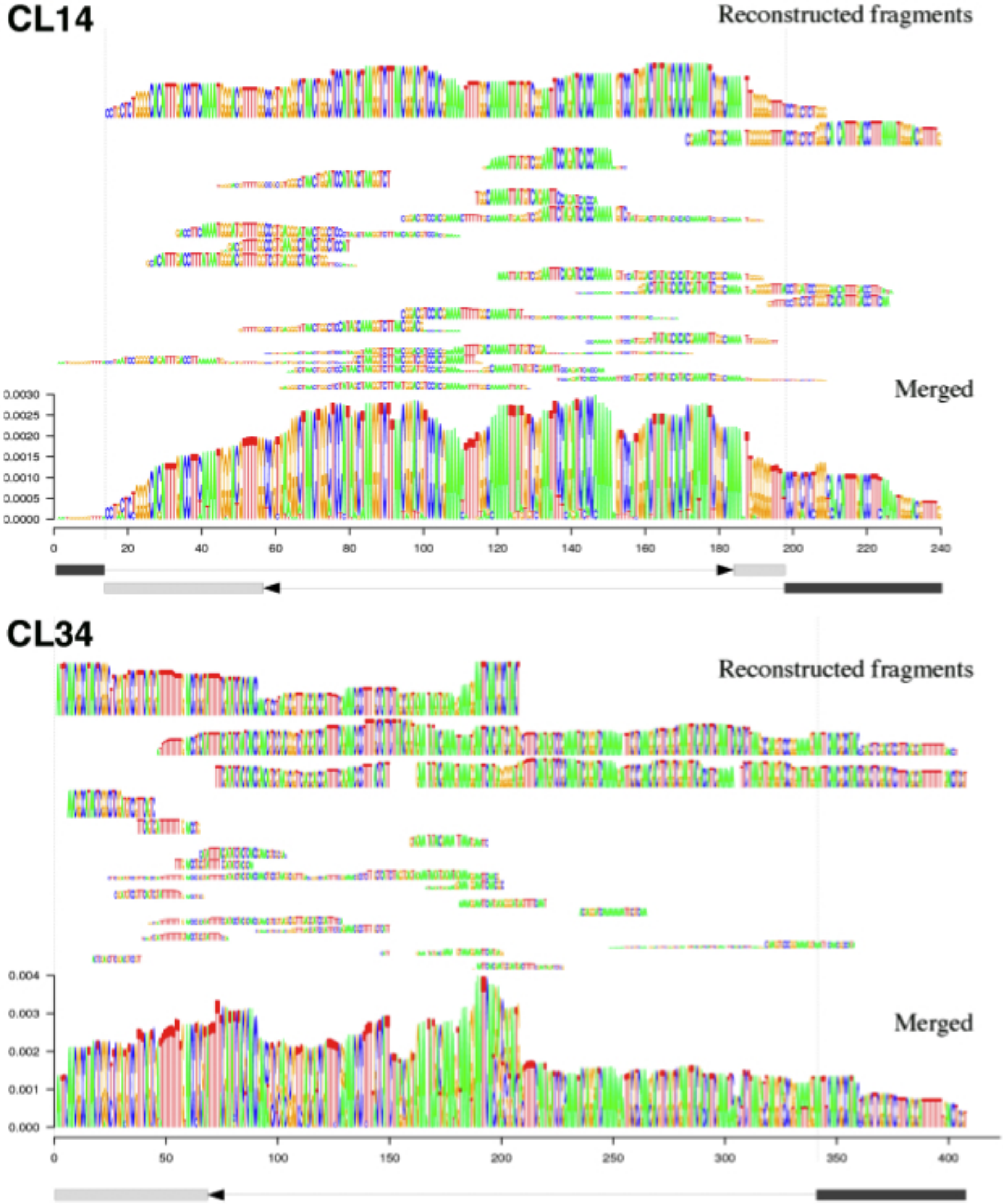

Figure S1 Mutual positions and sequences of fragments reconstructed from the most frequent k-mers used to build consensus sequences of the CL14 and CL34 repeats. Vertical lines indicate the beginning and end of the monomer. To produce the final logo, sequences extending outside the monomer region were moved and merged with the monomer logo as indicated below, and 1 bp insertions occurring at low frequencies were removed.

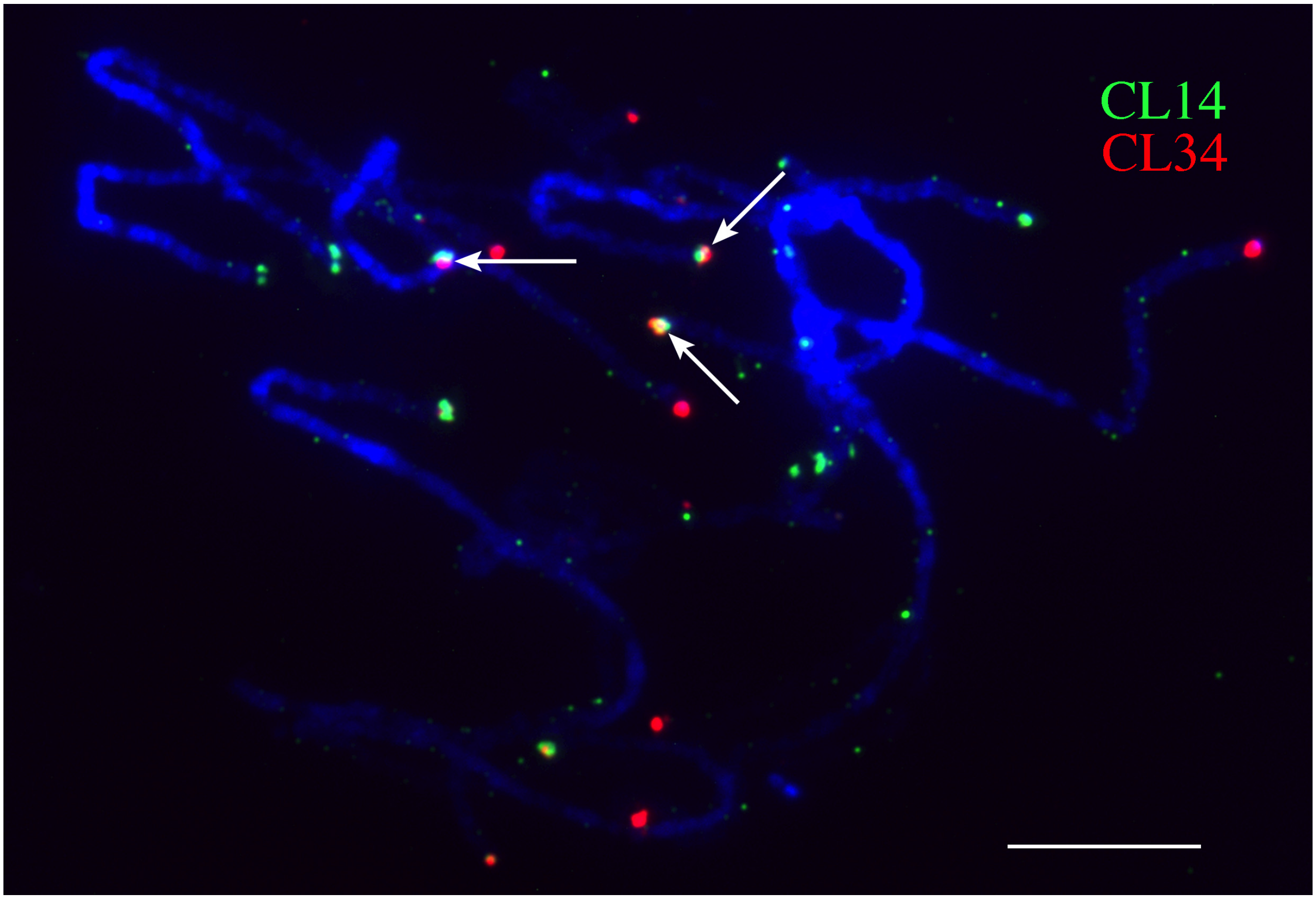

Figure S2 FISH mapping of CL14 (green) and CL34 (red) on pachytene chromosomes of DM1-3. Arrows point to the chromosomal ends where the CL34 signals are distal to the CL14 signals. Bar = 10 µm.
