# Supplementary material for: Organization and Evolution of Subtelomeric Satellite Repeats in the Potato Genome
Source: G3 (Bethesda). 2011 Jul 1;1(2):85–92. doi: 10.1534/g3.111.000125 (PMC3276127; doi:10.1534/g3.111.000125)
Supplement: Supporting Information [file supp_1.2.85_FigureS2.pdf]

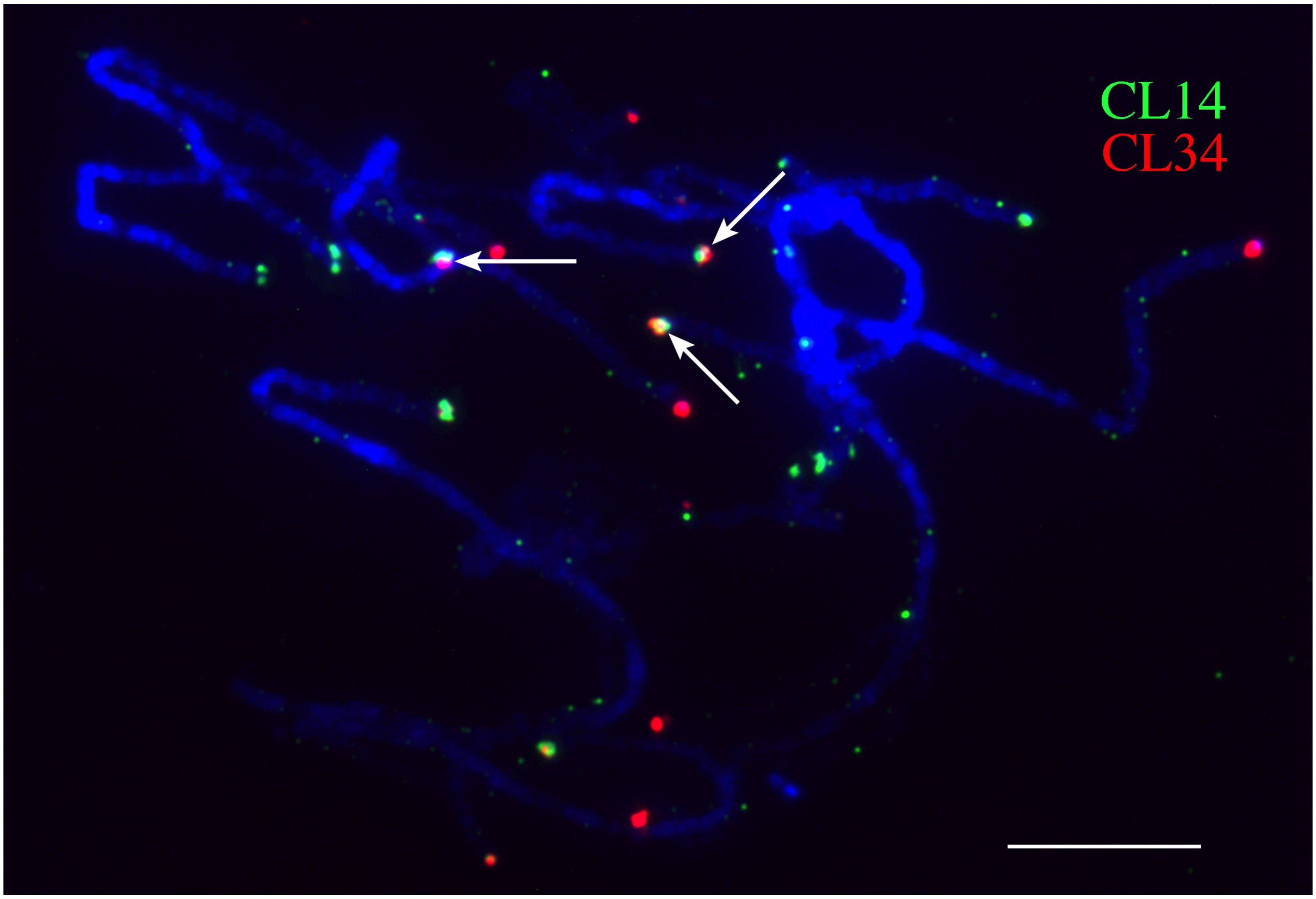

Figure S2 FISH mapping of CL14 (green) and CL34 (red) on pachytene chromosomes of DM1-3. Arrows point to the chromosomal ends where the CL34 signals are distal to the CL14 signals. Bar = 10 µm.
